# Combined Developmental Toxicity of the Pesticides Difenoconazole and Dimethomorph on Embryonic Zebrafish

**DOI:** 10.3390/toxins13120854

**Published:** 2021-12-01

**Authors:** Ruiqi Fan, Wanjun Zhang, Li Jia, Lizhong Li, Jun Zhao, Zengming Zhao, Shuangqing Peng, Yiqiang Chen, Xiaoyan Yuan

**Affiliations:** 1 Center of Disease Control and Prevention, PLA, Beijing 100073, China; fanruiqi94110@cau.edu.cn (R.F.); s20193040574@cau.edu.cn (W.Z.); acegi@163.com (L.J.); lilizhongali@126.com (L.L.); tangsong009@126.com (J.Z.); zhaozm000@163.com (Z.Z.); pengsq@hotmail.com (S.P.); 2 State Key Laboratory of Animal Nutrition, College of Animal Science and Technology, China Agricultural University, Beijing 100193, China; yqchen@cau.edu.cn; 3 School of Nursing and Health, Henan University, Kaifeng 475000, China

**Keywords:** difenoconazole, dimethomorph, combination, transcriptomics, environmental toxicity, pesticide

## Abstract

Difenoconazole (DIF) and dimethomorph (DIM) are widely used pesticides frequently detected together in environmental samples, so the deleterious effects of combined exposure warrant detailed examination. In this study, the individual and combined effects of DIM and DIF on conventional developmental parameters (hatching rate, deformity rate, lethality) and gene expression were measured in embryonic zebrafish. Both DIF and DIM interfered with normal zebrafish embryo development, and the most sensitive toxicity index for both was 96 h post-fertilization (hpf) deformity rate (BMDL_10_ values of 0.30 and 1.10 mg/L, respectively). The combination of DIF and DIM had mainly synergistic deleterious effects on 96 hpf deformity and mortality rates. Transcriptome analysis showed that these compounds markedly downregulated expression of mcm family genes, *cdk*1, and *cdc*20, thereby potentially disrupting DNA replication and cell cycle progression. Enhanced surveillance for this pesticide combination is recommended as simultaneous environmental exposure may be substantially more harmful than exposure to either compound alone.

## 1. Introduction

Difenoconazole (DIF) is a broad-spectrum triazole fungicide used globally to protect crops and seeds, and is generally considered safe for humans and mammals. However, recent studies have reported apparent toxic effects on aquatic animals such as *Daphnia magna* and zebrafish; the concentration of tested DIF ranged from 0.67 to 4.73 mg/L [1,2]. Besides, increased adult mortality, suppression of embryonic heart rate, and delayed embryonic development of DIF were also found; such effects can be seen within 0.18 to 1.47 mg/L [3]. These effects may result from dysregulation of genes controlling embryonic development and retinoic acid metabolism [4] as well as from the induction of cellular oxidative stress [5]. Residual DIF is often detected in crops as well as the surrounding soil, for example, 0.103–0.520 mg/kg in soil for mango in China, and 0.14–0.32 mg/kg in Italy [6,7]. Although there are four stereoisomers of difenoconazole, the toxicity data of different isomers are still lacking, and the existing reports temporarily do not consider the toxic effects of different isomers [8]. Given these reports of developmental toxicity, further studies are warranted on the dangers of DIF to ecosystems and human health.

Dimethomorph (DIM) is another broad-spectrum fungicide of the morpholine class with demonstrated efficacy against fungal spores. Thus, like DIF, DIM is used widely for the cultivation of fruits and vegetables. However, DIM residue has been detected in the surrounding environment of planted grapes within the range of 0.05–0.19 mg/kg [9], so the potential risks of DIM exposure should be evaluated. Also, considering the high rate of co-detection with DIF [10,11] and the potential for synergistic effects, comprehensive risk assessment requires evaluation of joint DIM plus DIF toxicity.

For instance, reports have confirmed that DIF, tebuconazole, and propiconazole in certain proportions can exert synergistic deleterious effects on zebrafish [3]. Similarly, diclofop-methyl and DIF can alter the biochemical parameters of oxidative stress in albino rats to a greater extent than equivalent concentrations of either compound alone [12]. However, the combined toxicity of DIF plus DIM exposure and the underlying mechanisms have not been studied. Transcriptomics is a valuable method for studying the underlying changes in gene expression associated with chemical toxicity, including the joint toxicity of various chemical combinations [13,14].

In this study, we analyzed the combined effects of DIF and DIM on zebrafish embryonic development and used transcriptomics to identify DEGs (differentially expressed genes). And qPCR method was implemented for validation of the genes altered by these compounds individually and in combination. These results suggest that DIF and DIM may have synergistic toxic effects, and their potential joint risks require more in-depth researches.

## 2. Results

### 2.1. Toxicity of DIF on Zebrafish Embryos

**Figure 1:**
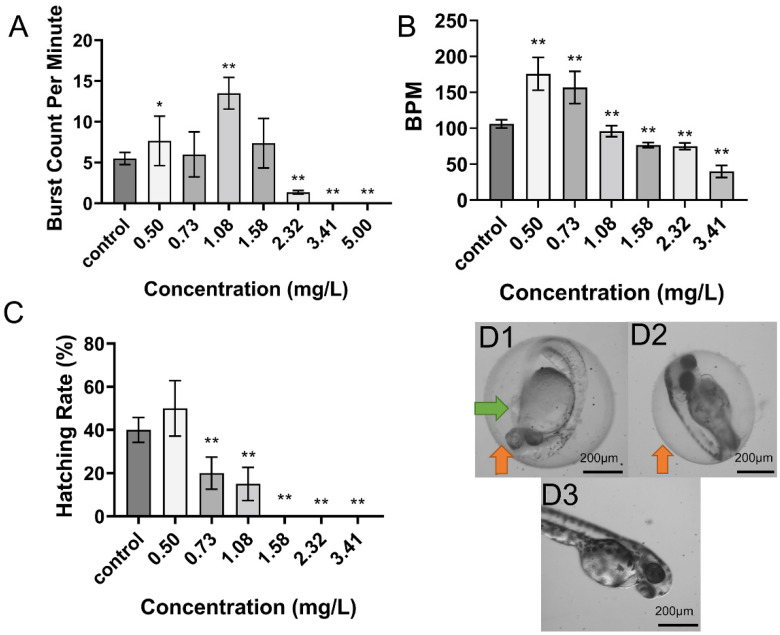
Effects of DIF on zebrafish embryo development. (**A**): Effect on tail flickstail flicks (bursts per min) at 24 hpf (*n* = 15 for each concentration). (**B**): Effect on larval heart rate (in beats per min [bpm]) at 48 hpf (*n* = 15 for each concentration). (**C**): Effect on hatching rate at 48 hpf (analyzed in triplicate). Data in (**A**–**C**) are shown as mean ± SEM; * *p* < 0.05, ** *p* < 0.01 [one-way ANOVA, Tukey’s Honestly Significant Difference test (Tukey’s HSD)]. D: Typical deformities caused by DIF at 48 hpf. (**D1**) 2.32 mg/L. (**D2**) 1.08 mg/L. (**D3**) 0.50 mg/L. Green arrows demarcate regions of yolk sac edema, and orange arrows indicate hatching delay.

DIFexposure had deleterious effects on multiple developmental parameters in zebrafish embryos (Figure 1), including LC_50_ at 96 hpf in 1.15 mg/L and EC_50_ of deformity at 96 hpf in 1.99 mg/L; all eggs were dead by 48 hpf at 5.00 mg/L. DIF also altered 24 hpf tail flicks and 48 hpf heart rate, with enhancement at low concentrations and inhibition at high concentrations (Figure 1A, B). In addition, DIF inhibited hatching rate at 48 hpf, with complete inhibition at doses ≥ 1.58 mg/L (Figure 1C). According to BMDL_10_ (Lower confidence limit of benchmark dose 10) values, the 96 hpf deformity rate was the most sensitive index of DIF toxicity (BMDL_10_ = 0.30 mg/L), followed by 48 hpf hatching rate (BMDL_10_ = 0.40 mg/L) and 48 hpf heart rate (BMDL_10_ = 0.54 mg/L), while 96 hpf lethality rate (BMDL_10_ = 0.71 mg/L) and 24 hpf autonomous movement (BMDL_10_ = 1.89 mg/L) were substantially less sensitive (see Table S2).

### 2.2. Toxicity of DIM on Zebrafish Embryo Development

**Figure 2:**
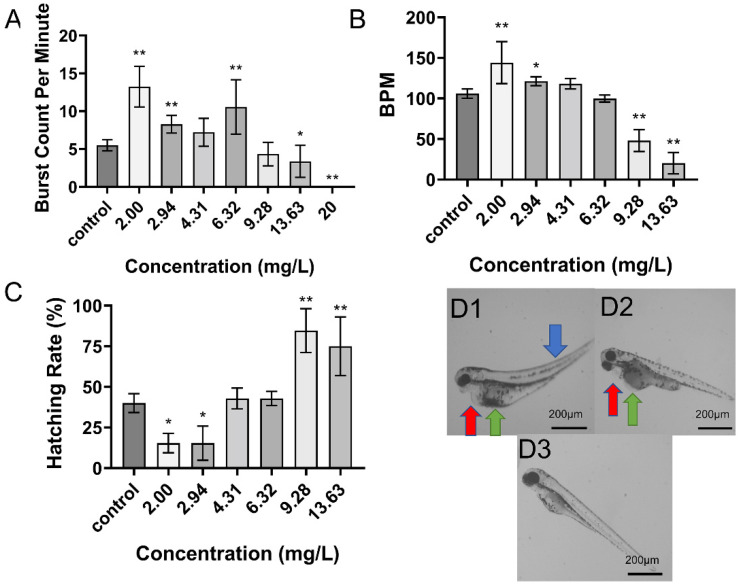
Effects of DIM on zebrafish developmental parameters. (**A**): Effect on tail flicks at 24 hpf (*n* = 15 for each concentration). (**B**): Effect on larval heart rate at 48 hpf (*n* = 15 for each concentration). (**C**): Effect on hatching rate at 48 hpf (analyzed in triplicate). Data in (**A**–**C**) are shown as mean ± SEM; * *p* < 0.05, ** *p* < 0.01 (ANOVA, Tukey’s HSD). D: Typical deformities caused by DIM at 48 hpf. (**D1**) 13.63 mg/L. (**D2**) 4.31 mg/L, (**D3**) 2.00 mg/L. Red arrows demarcate areas of pericardial edema, green arrows yolk sac edema, and blue arrows spinal curvature.

DIM exposure also had substantial deleterious effects on multiple developmental parameters in zebrafish embryos, although at generally higher concentrations than DIF (Figure 2). For instance, the 96 hpf EC_50_ of deformity and LC_50_ were 4.25 mg/L and 11.84 mg/L, respectively, markedly higher than the corresponding values for DIF. Besides, all eggs were dead by 48 hpf at the highest concentration. Like DIF, DIM also enhanced 24 hpf autonomous movement and 48 hpf larvae heart rate at low concentrations but inhibited these metrics at higher concentrations. Conversely, DIM inhibited 48 hpf hatching rate at low concentrations (2.00–2.94 mg/L) and enhanced hatching rate at high concentrations (Figure 2C). According to BMDL_10_ values, 96 hpf deformity rate was the most sensitive index of developmental toxicity (BMDL_10_ = 1.10 mg/L), followed by 48 hpf heart rate (BMDL_10_ = 2.31 mg/L), 96 hpf lethality rate (4.10 mg/L), 48 hpf hatching rate (5.92 mg/L), and 24 hpf tail flicks (7.90 mg/L) (Supplementary Materials Table S2). In general, DIM alone was less toxic to zebrafish embryos than DIF, and the BMDL_10_ value of the most sensitive index was about 3.5 times higher than the corresponding value for DIF.

### 2.3. Toxicity of Combined Exposure to DIF and DIM

**Figure 3:**
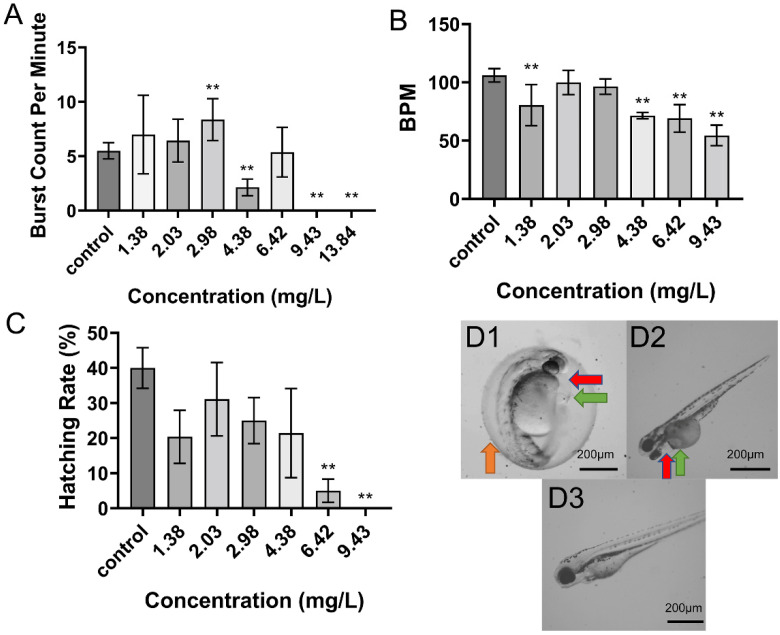
Effects of combined DIF and DIM exposure (MIX) on developmental parameters. (**A**): Effect on tail flicks at 24 hpf (*n* = 15 for each concentration). (**B**): Effect on larval heart rate at 48 hpf (*n* = 15 for each concentration). (**C**): Effect on hatching rate at 48 hpf (analyzed in triplicate). Data in (**A**–**C**) are shown as mean ± SEM; ** *p* < 0.01 (ANOVA, Tukey’s HSD) D: Typical deformities caused by MIX at 48 hpf. (**D1**) 4.38 mg/L. (**D2**) 2.03 mg/L. (**D3**) 1.38 mg/L. Red arrows demarcate areas of pericardial edema, green arrows demarcate areas of yolk sac edema, and orange arrows indicate hatching delay.

Combined exposure to both pesticides also induced significant dose-dependent reductions in 24 hpf tail flicks, 48 hpf heart rate, and 48 hpf hatching rate (Figure 3A–C). The dose–deformity and dose–lethality curves yielded a 96-hpf EC_50_ of deformity of 3.47 mg/L and a 96-hpf LC_50_ of 4.20 mg/L, respectively. All eggs were dead by 48 hpf at the highest concentration so that the data of 48-hpf heart beat at 13.63 mg/L was missing. Unlike single pesticide exposure, the most sensitive indicator of mixed exposure was 48 hpf heart rate (BMDL_10_ = 0.18 mg/L), followed by 48-hpf hatching rate (BMDL_10_ = 1.37 mg/L), 96 hpf lethality rate (1.55 mg/L), 96 hpf deformity rate (2.74 mg/L), and 24 hpf tail flicks (5.90 mg/L) (Supplementary Materials Table S2). The 48 hpf heart rate was a markedly more sensitive index of toxicity under combined treatment than under treatment with either compound alone.

### 2.4. Synergistic Effects of Combined DIF and DIM on Developmental Parameters

**Figure 4:**
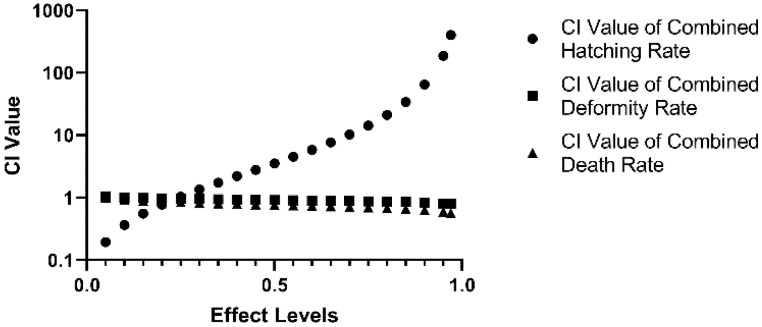
Combination index (CI) values of MIX for hatching rate (circle), deformity rate (square), and death rate (triangle).

Combination indices (CI values) were calculated from single-drug and mixed exposure data to assess concentration-dependent antagonistic, additive, and synergistic interactions of DIF and DIM. As shown in Figure 4, the two compounds exhibited synergy on hatching rate at low effect levels (0–20%) as indicated by CI values < 0.9 but antagonism at effect levels > 20% as indicated by CI values > 1.1. The CI values of MIX for deformity and lethality rates were within the additive CI range at low effect levels but indicated mild synergy at high effect levels (15–100% for combined death rate, 55–100% for combined deformity rate).

### 2.5. Transcriptomic and qPCR Analyses of Differentially Expressed Genes under DIF, DIM, and Combined Treatment

**Figure 5:**
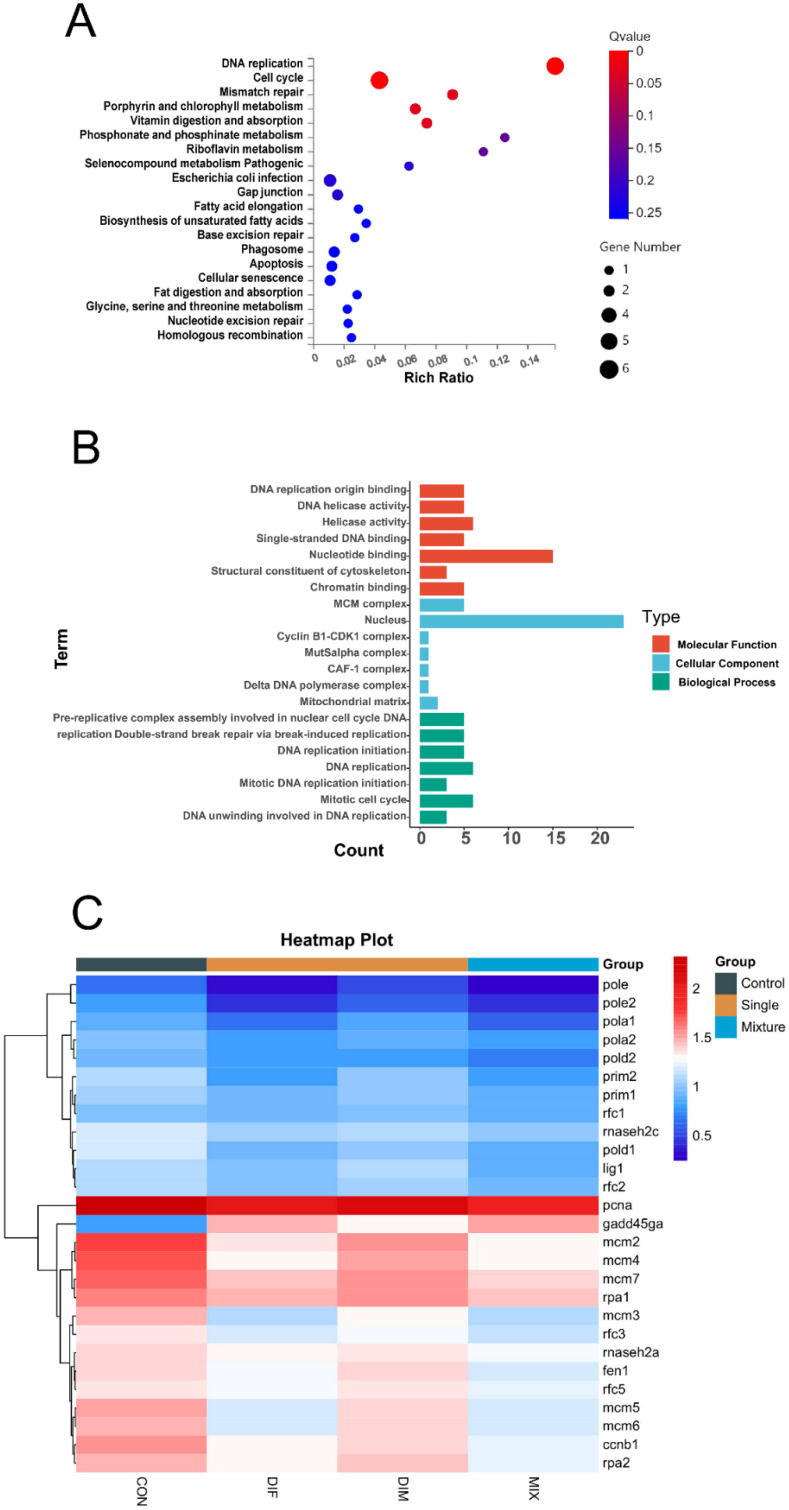
Differentially expressed genes (DEGs) in zebrafish embryos following DIF, DIM, and combined treatment. (**A**): KEGG pathway enrichment of common differentially expressed genes in DIF, DIM, and MIX. (**B**): GO enrichment of common DEGs in DIF, DIM, and MIX. (**C**): Heat map of top DEGs.

The results of RNA-Seq revealed 4409 differentially expressed genes (DEGs) following DIF treatment, including 1749 upregulated and 2660 downregulated genes. In comparison, DIM induced relatively few DEGs, with only 101 upregulated and 74 downregulated genes. In the MIX treatment group, 1322 DEGs were upregulated and 2000 were downregulated. We then focused on the 79 genes modulated by both single exposure and mixed exposure given the synergist effects of these two compounds on deformity and mortality rates. The DEGs common in the DIF, DIM, and MIX groups were enriched, and then compared to the Kyoto Encyclopedia of Genes and Genomes (KEGG) pathways. ‘DNA replication’ and ‘cell cycle mismatch repair’ were the most enriched pathways (Figure 5A), including the mini-chromosome maintenance (mcm) family genes *mcm*2, *mcm*3, *mcm*4, and *mcm*6, the cell cycle regulators *cdk*1 and *cdc*20, and the mismatch repair genes msh6 and pold1. Gene Ontogeny (GO) analysis also revealed that the DEGs common in the DIF, DIM, and MIX groups were enriched in genes associated with ‘DNA replication’, ‘MCM protein function’, and other aspects of biological function (Figure 5B).

**Figure 6:**
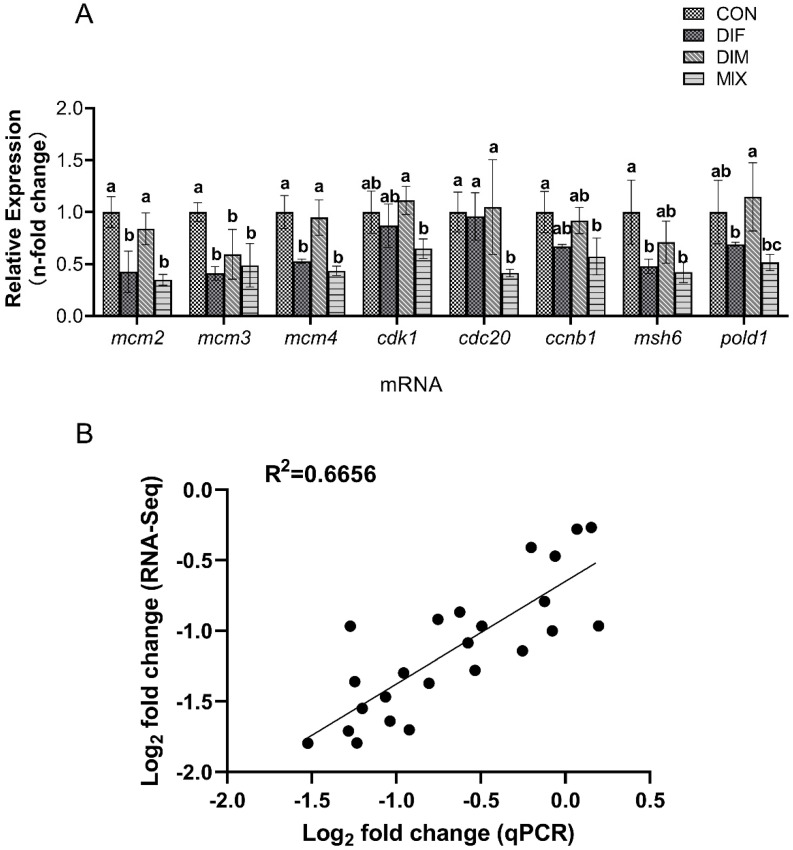
Validation of transcriptomic by qPCR. Genes related to cell cycle and DNA replicate were selected for the validation by using qPCR. (**A**): Relative mRNA expression levels of genes differentially modulated by DIF, DIM, and the combination (MIX). All samples were extracted and analyzed in triplicate. Data are shown as mean ± SEM; lowercase letters were used to reveal the differences, completely different lowercase letters above the bars indicate significant differences (*p* < 0.05), while any of the same lowercase letters indicate no significant difference (*p* > 0.05). (**B**): The correlation between transcriptomic analysis and qPCR, the higher R square value reveals the greater correlation. The X-axis represents log2-fold change in the expression levels found by qRT-PCR. The Y-axis represents the log2 value of the expression level fold change from RNA-Seq.

The identities of shared DEGs were further confirmed by qPCR. Both DIF and DIM as well as the combination (MIX group) downregulated the expression levels of *mcm*2, *mcm*3, and *mcm*4. While DIM alone had no effect on the expression levels of *cdk*1 and *cdc*20, it potentiated the downregulation of both genes induced by DIF. Moreover, DIF and DIM also synergistically downregulated multiple genes related to DNA replication and cell cycle control, such as ccnb1, msh6, and pold1 (Figure 6A). The correlation between qPCR and RNA-Seq was also checked, and the Pearson R square was 0.6656, which indicated a strong correlation (Figure 6B).

## 3. Discussion

Widespread application of DIFand DIM to protect crops has increased the risks of mixed environmental contamination and simultaneous exposure. Residual concentrations of DIF have been detected in both soil and water samples from India and Brazil, the concentrations detected can be up to 35 μg/L in irrigation water and 1.5 mg/kg in soil [15,16]. In some provinces of China, DIF concentrations as high as 1.00–2.36 mg/kg have been measured in paddy water [17], which is even higher than the 96 hpf LC50 for zebrafish embryos measured in the present study. Similarly, DIM concentrations from 0.20 mg/kg [18] to 0.70 mg/kg [9] have been reported. Further, DIM has a long elimination half-life in soil [19] and can enter groundwater [20] through leaching. Thus, environmental and health risk assessments are warranted.

Calculation of the combination index (CI) for DIF plus DIM (MIX) revealed synergistic effects on mortality and teratogenicity (CI < 0.9) within the high-dose effect range. In addition, the BMDL_10_ values of MIX for tail flicks and heart rate were lower than the weighted averages of the individual-agent BMDL_10_ values, suggesting that the risk threshold concentration for mixed exposure may be lower than that of individual exposure. EuroMix and other projects focusing on the health risks of multi-component pollutant exposure [21] have found several examples of increased risk from combined pollutants compared to the individual components [22]. The findings of this study suggest that DIF and DIM may have a synergistic toxic effect and more researches are needed for environmental health risk assessment of combined exposure to these two pesticides.

The toxicity of DIF has been extensively investigated. Zhu et al. found that DIF caused cardiotoxicity by inducing oxidative stress and apoptosis in early life stages of zebrafish within 0.3–1.2 mg/L [5], while Teng et al. found that DIF altered amino acid and lipid metabolism in early-stage zebrafish within 0.5–500 μg/L [23]. In contrast, we found that DIF reduced the expression levels of multiple genes related to DNA replication and cell cycle regulation, potentially accounting for the significant disruption of embryonic development and survival. Teng et al. reported parental exposure to DIF at environmentally relevant exposure concentrations could induce detrimental response in the offspring by disruption of sex steroid hormones and vitellogenin [24]. Therefore, it is possible that adverse response is ultimately impaired by a combination of oxidative stress, dysregulation of cell proliferation, and aberrant endocrine signaling.

In contrast to DIF, there have been relatively few studies on DIM toxicity. Regueiro et al. reported cytotoxicity against primary cultured cortical neurons after exposure to 0.1–100 µM DIM for 7 days [25], providing a potential explanation for the observed disruptions in zebrafish motor activity and heart rate regulation. The overall developmental toxicity of DIM was lower than that of DIF, suggesting less risk from DIM exposure alone. However, analysis of combined exposure suggested that DIM may amplify DIF developmental toxicity.

DNA replication influences all aspects of development and maturation. In zebrafish, about 20% of all cells are undergoing DNA replication at 6 hpf [26]. Thus, the effects of DIF and DIM on DNA replication may disrupt all subsequent stages of development. Similarly, cell cycle regulation is critical during early development. For instance, the developmental toxicity of silver nanoparticles was attributed to cell cycle arrest and upregulation of genes such as ccna1 and stag3 [27], which suggests that the cell cycle affected by chemicals may attribute to the toxicity effects

Genes of the *mcm* family were among the most frequently and severely dysregulated by DIF, DIM, and MIX. These genes encode minichromosome maintenance (MCM) proteins that control DNA replication by regulating checkpoint signaling pathways [28]. MCM proteins are also markers of proliferation zones during embryogenesis [29], so reduced function concomitant with gene downregulation may disrupt the proper patterning of cell amplification. Hou et al. found that the toxicity of Nano-ZnO on zebrafish was associated with effects on the expression levels of *mcm* family genes [30], which suggests that the *mcm* genes may be related to the adverse effects of chemicals on zebrafish. Moreover, *mcm* genes are involved in large regulatory networks, so both DIF and DIM may have broader biological effects that warrant further investigation.

In addition to *mcm* genes, both DIM and DIF altered expression levels of the cell cycle regulatory genes cyclin-dependent kinase 1 (cdk1) and cell division cycle protein 20 (cdc20). The cell cycle is tightly regulated to promote correct morphological development during growth [31]. In addition to malformation, dysregulation of the cell cycle and related functions may cause cell proliferation to stagnate and increase mortality [32].

Inhibition of CDK1 may enhance cellular sensitivity to DNA damage [33]. Li et al. also found that the fungal toxin zearalenone damaged porcine granulosa cells by altering the expression of *cdk*1 and related genes [34]. In addition, the pesticide glyphosate altered CDK1 function in sea urchin embryonic cells [35]. Thus, disruption of normal *cdk*1 expression may contribute to the toxicity of many distinct compounds. Similarly, dysregulation of *cdc*20 expression may induce cell damage. Mansur et al. found that bisphenol A significantly inhibited CDC20 expression in human cumulus cells [36]. Chou et al. also found that the toxic effect of phenethyl isothiocyanate against human glioblastoma GBM 8401 cells was associated with CDC20 downregulation [37]. Although there have been a number of reports on chemical toxicity mediated by dysregulation of *cdk*1 or *cdc*20, all were in vitro models. Here we demonstrate that DIM and DIF can also disrupt the regulation of *cdk*1 and *cdc*20 expression levels in zebrafish embryos, which may in turn contribute to the developmental toxicity of these compounds.

Although the expression of most genes was within two-fold change compared to the control group, the selected genes showed a strong correlation with the results of the transcriptome (Pearson R^2^ = 0.6656), indicating these sites in DNA replication and cell cycle related pathways had been affected. Considering that the dose selected in the experiment was BMDL_10_, a point of departure, the toxic effect may not be relatively obvious. Therefore, it was a possible phenomenon that the fold-change of mRNA expression was within 2. Similar results can be found in several studies [38,39]. In summary, *mcm* family genes, *cdk*1 and *cdc*20 may serve as sensitive sites for the embryonic developmental toxicity of DIFand DIM, but more dose-effect experiments are needed to explore further.

## 4. Conclusions

This study confirmed that DIF could affect the developmental parameters of zebrafish within the range of 0.50–5.00 mg/L, and DIM could also affect developmental parameters of zebrafish within the range of 2.00–20.00 mg/L. The combination of DIF and DIM had mainly synergistic deleterious effects on 96 hpf deformity and mortality rates. Transcriptome analysis showed that these compounds markedly downregulated expression of *mcm* family genes, *cdk*1, and *cdc*20, thereby potentially disrupting DNA replication and cell cycle progression. These findings also further highlight the utility of transcriptomics to reveal potential mechanisms underlying the synergistic effects of various toxin combinations. More extensive surveillance of this fungicide combination in agriculture and environment samples appears warranted for comprehensive risk assessment of DIF and DIM exposure.

## 5. Materials and Methods

### 5.1. Reagents

Difenoconazole (purity: 99.9%, CAS Number: 119446-68-3) and dimethomorph (purity: 98.7%, CAS Number: 110488-70-5) were purchased from Dr. Ehrenstorfer GmbH (Augsburg, Germany), and DMSO (purity: 99.7%, LC-MS Grade) was purchased from Thermo Fisher (Waltham, MA, USA). Other reagents (analytical grade) were purchased from Shanghai Sinopharm Chemical Reagent (Shanghai, China).

### 5.2. Maintenance and Breeding of Zebrafish

Use of Laboratory Animals and approved by the Animal Welfare and Animal Experimental Ethical Committee at the China Agricultural University (Beijing, China) (certification no: AW11111208–1–1). Wild-type Tu zebrafish were purchased from Wuhan Zebrafish Resource Center (Wuhan, China) and housed in a dedicated aquatic system (ESEN Technology, Beijing, China). The light and dark cycle was controlled at 14 h/10 h and room temperature at 28 ± 2 °C. Fish were fed freshly hatched brine shrimp larvae twice daily. In the afternoon before experiments, two male and two female fish were placed in separate compartments of a breeding box. At the beginning of the photoperiod the following day, the partitions were removed to allow the males and females to chase freely. After mating, the fertilized eggs were collected within 30 min. To remove unfertilized, coagulated, and damaged eggs, collected eggs were examined under an SZ-10 stereomicroscope (Olympus, Japan). Then, the eggs were kept at 28 °C until the experiment began.

### 5.3. Pesticide Exposure

Pesticides were weighed, dissolved in LC-MS grade DMSO as stock solutions, and diluted in Holt buffer (containing NaCl 3.5 g/L, KCl 0.05 g/L, CaCl_2_ 0.1 g/L, NaHCO_3_ 0.05 g/L) to the indicated treatment concentrations. The final concentration of DMSO was 0.1%, which had no effects on developmental parameters in preliminary experiments. The final DIF concentrations were 0.50, 0.73, 1.08, 1.58, 2.32, 3.41, and 5.00 mg/L, and the final DIM concentration were 2.00, 2.94, 4.31, 6.32, 9.28, 13.63, and 20.00 mg/L. The mixed exposure liquid MIX (DIF: DIM = 1:5.92, *v*/*v*) was prepared according to the principle of equipotent concentration ratios. The total concentrations of MIX were 1.38, 2.03, 2.98, 4.38, 6.42, 9.43, and 13.84 mg/L.

The exposure started at 4 hpf. We referred to the exposure design of Park et al. [40]. For treatment, 20 healthy eggs were placed in each well of a 6-well plate and treated with 10 mL of the indicated solution, with 3 replicates at each DIF, DIM, or MIX concentration. The solution was renewed every 24 h for 96 h and dead eggs were removed at each solution exchange. Embryonic mortality and malformation rates of each treatment group were recorded at 96 hpf.

### 5.4. Measurement of Developmental Parameters

Mortality as evidenced by egg coagulation or cardiac arrest was determined at 24, 48, 72, and 96 hpf. At the same time, the rate of deformity (including pericardial edema, yolk sac edema, and tail flicks) was calculated for each treatment group at 48, 72, and 96 hpf, the temperature was kept at 28 ± 2 °C during all measurements. The hatching rate, defined as total separation of juvenile fish from the egg membrane, was calculated at 48 hpf. Five embryos in one replicate were selected and recorded under a stereoscopic microscope for 1 min tail flicks at 24 hpf and for 30-s heart rate at 48 hpf using MediaRecorder4.0 (Noldus, Wageningen, The Netherlands). We considered a tail flick as a movement more significant than 15 pixels recorded by the software. The heart rate values were multiplied by 2 and converted to beats per minute. Video records were digitized for analysis using DanioScope 1.1 (Noldus, Wageningen, The Netherlands). The effects of pesticide exposure are expressed as10% benchmark dose levels (BMDL_10_ values). For tail flicks and heart beat measurements, only alive eggs or larvae were tested. The hatching rates were calculated by dividing the number of hatched eggs by the number of all the alive eggs and larvae.

### 5.5. Transcriptomics

For gene expression analyses, fertilized eggs were treated as indicated starting at 4 hpf until 96 hpf, with 3 biological replicates per treatment group and 80 eggs in each replicate. After 96 hpf, 40 fertilized eggs of each replicate were transferred to a 2-mL centrifuge tube, and the supernatant separated by centrifugation at 12,000× *g* and 4 °C. Eggs were then washed twice with 1 mL 1 × phosphate-buffered saline (PBS) with centrifugation after each wash to remove the supernatant. Total RNA was extracted from washed eggs using Trizol according to the manufacturer’s instructions (Ambion, Waltham, MA, USA) and stored at −80 °C until transcriptomics analysis using BGI-Seq (see detailed protocol in the Supplementary Materials). The transcriptome raw data have been submitted to NCBI database with the BioProject number PRJNA780940.

### 5.6. Real-Time Quantitative Reverse Transcription PCR

RNA was extracted from the remaining eggs of each replicate using Trizol as described and reverse transcribed using a commercial kit (Thermo Scientific, Waltham, MA, USA). Gene expression levels were estimated using an LightCycler480 II thermocycler and 2X SG Fast qPCR Master Mix (Roche, Basel, Switzerland), and the primer sequences listed in the Supplementary Materials. The reaction volume was 25 μL, and the thermocycle protocol was 95 °C for 3 min as initial step, 45 cycles of 95 °C for 5 s, 60 °C for 30 s, and dissociated according to instrument guidelines. Each treatment was analyzed in 3 biological replicates and each sample was tested in 3 technical replicates. Relative gene expression levels were calculated using the 2^−ΔΔCT^ method and β-actin as the reference gene (see primer pairs of selected genes in Table S1)

### 5.7. Statistics

All developmental parameters and LC50 values were calculated and compared using SPSS 22.0. Developmental parameters and qPCR analysis results were expressed as mean ± standard deviation. One-way analysis of variance (ANOVA) and Tukey’s test were used to compare the means of multiple groups if they passed Levene’s Test. *p*-values < 0.05 were considered to be statistically significant. Graphpad 8.0 was used to draw 96 hpf LC curves and BMDS 3.2 software to calculate 10% benchmark dose levels (BMDL_10_ values) [41]. CompuSyn 1.0.4 software [42] was used to constructe 96 hpf concentration-lethality and concentration-deformation rate curves, and to calculate the combination index (CI) values for each developmental parameter. Transcriptomic results were analyzed using the BGI Dr.Tom online work platform (https://biosys.bgi.com, accessed on 1 October 2021).

## Data Availability

Data will be available with permission of Ministry of Science and Technology of the People’s Republic of China.

## Supplementary Materials

The following are available online at https://www.mdpi.com/article/10.3390/toxins13120854/s1, Table S1: Primer pairs of selected genes in qRT-PCR analysis; Table S2: BMDL_10_ of developmental parameters after exposure of difenoconazole, dimethomorph and mixture of the two; Table S3: Data generated by the zebrafish transcriptome and quality filtering; Table S4: Significantly changed KEGG pathways and related genes; Table S5: GO categories of the differentially expressed genes.
