# Supplementary material for: Combined Developmental Toxicity of the Pesticides Difenoconazole and Dimethomorph on Embryonic Zebrafish
Source: Toxins (Basel). 2021 Dec 1;13(12):854. doi: 10.3390/toxins13120854 (PMC8706556; doi:10.3390/toxins13120854)
Supplement: Supplementary file 1 [file toxins-13-00854-s001.zip › toxins-1454757.pdf]

Supplementary Materials: Combined Developmental Toxicity of the Pesticides Difenoconazole and Dimethomorph on Embryonic Zebrafish

Ruiqi Fan, Wanjun Zhang, Li Jia, Lizhong Li, Jun Zhao, Zengming Zhao, Shuangqing Peng, Yiqiang Chen and **Xiaoyan Yuan**

1. mRNA Library Construction

Firstly, RIN (RNA integrity number) and concentration were assessed using Fragment Analyzer 5400 (Agilent, USA), the RIN number should be over 8. Oligo(dT)-attached magnetic beads were then used to purify mRNA. The upcoming step was to generate the first-strand cDNA, which through a random hexamer-primed reverse transcription. Second-strand cDNA synthesis came next. The final library was formatted by single-strand circle DNA (ssCir DNA) The final library was amplified with phi29 to make DNA nanoballs (DNB), with more than 300 copies of one molecular. DNBs were loaded into the patterned nanoarray, and single-end 50 bases reads were generated on the BGIseq500 platform (BGI-Shenzhen, China).

2. RNA-Seq Data Analyze

The filter of the sequencing data was with SOAPnuke [1]. We define reads that contain more than 20% of quality ≤ 5-base as low-quality reads and define reads whose unknown base ('N' base) ratio is more than 5% as unknown reads. Then, low-quality and unknown reads were removed. Details of filtered reads can be found in Supplementary Table 3. The remained clean reads were obtained and stored in FASTQ format. According to the official documents, we used HISAT2 (v2.0.4) to map the clean reads to the reference genome [2]. The reference database was provided by NCBI (GRCz11). The total mapping percentage ranged from 87.13% to 91.05%. Bowtie2 (v2.2.5) was applied to align the clean reads to the reference coding gene set [3], and the mapping percentage ranged from 64.30%-77.66%. RSEM (v1.2.12) was used to calculate the expression level of genes [4]. DESeq2(v1.4.5) was then used to analyze the differential expression levels [5]. The selected genes were transferred to Gene ID and then compared to the GO (http://www.geneontology.org/, accessed on 13 October 2020) and KEGG (https://www.kegg.jp/, accessed on 13 October 2020) database, then performed the enrichment analysis of annotated different expressed genes, results contained gene numbers and enrichment factor (See Supplementary Table 4 and 5). The significant terms and pathways were corrected by Q value with a rigorous threshold (Q value ≤ 0.05) [6]. The heatmap and GO network were completed through Hiplot (https://hiplot.com.cn/, accessed on 13 October 2020).

SUPPLEMENTARY TABLES

Supplementary Table S1: Primer pairs of selected genes in qRT-PCR analysis

Gene	Forward Primer	Reverse Primer
β-actin	TCAGTGCACGCTGAGAAGAT	ATGCCAACCATCACTCCCTGA
mcm2	TTCGATCACTCAGGCAACTCC	GATGCGCTGGTAGTTCTGGT
mcm3	CTCAAGACACACCACGACCA	CCGGAGACTGAATCTGTGGA
mcm4	CAGTTCACAGACCCCGACTC	TGGGGATGACTTCCTGTGGA
cdk1	CACCCAAACGTTGTACGCCTG	TTCGCCTGATGGGATGGAGT
cdc20	AAAACATTGTTGGAATTGTGTTACC	AGGGGGTGTTTTGGGTCTTG
ccnb1	CGTGTCACAAGGAACACTCG	CCTGTCGTGTTTGCGGATTG
msh6	GCATGGCAGTGCTCGATGTG	GTGACACAAGGGTGACGAGA
pold1	CTGCCCCTAACGGGTTTACG	GAGCGCATGTCCTTCAACAC

Supplementary Table S2: BMDL10 of developmental parameters after exposure of difenoconazole, dimethomorph and mixture of the two

**Parameters**	**DIF**		**DIM**		**MIX**	
	**Best-fitted model**	**$BMDL_{10}$**	**Best-fitted model**	**$BMDL_{10}$**	**Best-fitted model**	**$BMDL_{10}$**
**24 hpf autonomous tail swinging**	Exponential degree 3	1.89	Exponential degree 3	7.90	Exponential degree 3	5.90
**48 hpf heart rate**	Hill	0.54	Exponential degree 3	2.31	Exponential degree 4	0.18
**48 hpf hatching rate**	Exponential degree 3	0.40	Hill	5.92	Polynomial degree 5	1.37
**96 hpf death rate**	Exponential degree 5	0.71	Hill	4.10	Exponential degree 5	1.55
**96 hpf deformity rate**	Hill	0.30	Exponential degree 5	1.10	Hill	2.74

Supplementary Table S3: Data generated by the zebrafish transcriptome and quality filtering

Sample Name	Group	Total Bases Count (bp)	Total Bases Count (Gb)	Total Clean Bases (Gb)	Q20 Bases Ratio (%)	Q30 Bases Ratio (%)	Clean Reads Ratio (%)
con-1	CON	45.57	44.41	6.66	97.25	92.68	97.45
con-2		45.57	44.32	6.65	97.38	93.03	97.26
con-3		45.57	44.09	6.61	97.27	92.76	96.73
dif-1	DIF	45.57	43.94	6.59	97.35	92.97	96.41
dif-2		45.57	44.07	6.61	97.31	92.84	96.69
dif-3		45.57	44.25	6.64	97.3	92.83	97.09
dim-1	DIM	45.57	44.25	6.64	97.22	92.62	97.1
dim-2		47.33	44.59	6.69	97.42	93.16	94.22
dim-3		47.33	45.5	6.83	97.31	92.92	96.15
mix-1	MIX	45.57	44.26	6.64	97.32	92.92	97.13
mix-2		45.57	43.93	6.59	97.42	93.12	96.4
mix-3		45.57	44.16	6.62	97.35	92.94	96.89

Supplementary Table S4: Significantly changed KEGG pathways and related genes

Pathway ID	Pathway	Number of DEGs in the pathway	Rich ratio	Q Value	Related genes (Gene ID)
3030	DNA replication	6	0.157895	3.36×10^{-8}	mcm3 (ENSDARG00000024204)<br>mcm2 (ENSDARG00000102798)<br>mcm5 (ENSDARG00000019507)<br>mcm4 (ENSDARG00000040041)<br>pold1 (ENSDARG00000027689)<br>mcm6 (ENSDARG00000057683)
4110	Cell cycle	6	0.043165	4.53×10^{-5}	mcm3 (ENSDARG00000024204)<br>mcm2 (ENSDARG00000102798)<br>mcm5 (ENSDARG00000019507)<br>mcm4 (ENSDARG00000040041)<br>mcm6(ENSDARG00000057683)<br>ccnb1(ENSDARG00000051923)
3430	Mismatch repair	2	0.090909	0.025766	msh6 (ENSDARG00000104541)<br>pold1 (ENSDARG00000027689)
860	Porphyrin and chlorophyll metabolism	2	0.066667	0.0287	cpox (ENSDARG00000062025)<br>alas1 (ENSDARG00000021059)
4977	Vitamin digestion and absorption	2	0.074074	0.0287	apoa4b.3 (ENSDARG00000094929)<br>cubn (ENSDARG00000087013)
440	Phosphonate and phosphinate metabolism	1	0.125	0.15703	pcyt1bb (ENSDARG00000104207)
740	Riboflavin metabolism	1	0.111111	0.15703	acp5a (ENSDARG00000019763)
450	Selenocompound metabolism	1	0.0625	0.215012	sephs1 (ENSDARG00000058292)
5130	Pathogenic Escherichia coli infection	3	0.010909	0.215012	tuba1b (ENSDARG00000045367)<br>myo1d (ENSDARG00000036863)<br>tuba1a (ENSDARG00000001889)
4540	Gap junction	2	0.015873	0.219258	tuba1b (ENSDARG00000045367)<br>tuba1a (ENSDARG00000001889)
62	Fatty acid elongation	1	0.029412	0.255652	hacd2 (ENSDARG00000014806)
1040	Biosynthesis of unsaturated fatty acids	1	0.034483	0.255652	hacd2 (ENSDARG00000014806)
3410	Base excision repair	1	0.027027	0.255652	pold1 (ENSDARG00000027689)
4145	Phagosome	2	0.013699	0.255652	tuba1b (ENSDARG00000045367)<br>tuba1a (ENSDARG00000001889)
4210	Apoptosis	2	0.012048	0.255652	tuba1b (ENSDARG00000045367)<br>tuba1a (ENSDARG00000001889)
4218	Cellular senescence	2	0.010929	0.255652	mybl2b (ENSDARG00000032264)<br>ccnb1 (ENSDARG00000051923)
4975	Fat digestion and absorption	1	0.028571	0.255652	apoa4b.3 (ENSDARG00000094929)
260	Glycine, serine and threonine metabolism	1	0.022222	0.257217	alas1 (ENSDARG00000021059)
3420	Nucleotide excision repair	1	0.022727	0.257217	pold1 (ENSDARG00000027689)
3440	Homologous recombination	1	0.025	0.257217	pold1 (ENSDARG00000027689)

Supplementary Table S5: GO categories of the differentially expressed genes

Category	Term (GO ID)	Number of genes	Rich ratio	Q Value	Related genes (Gene ID)
	**Molecular function**				
	DNA replication origin binding (GO:0003688)	5	0.263158	4.65×10^{-7}	mcm3 (ENSDARG00000024204)<br>mcm2 (ENSDARG00000102798)<br>mcm5 (ENSDARG00000019507)<br>mcm4 (ENSDARG00000040041)<br>mcm6 (ENSDARG00000057683)
	DNA helicase activity (GO:0003678)	5	0.119048	1.60×10^{-5}	mcm3 (ENSDARG00000024204)<br>mcm2 (ENSDARG00000102798)<br>mcm5 (ENSDARG00000019507)<br>mcm4 (ENSDARG00000040041)<br>mcm6 (ENSDARG00000057683)
	Helicase activity (GO:0004386)	6	0.066667	2.13×10^{-5}	mcm3 (ENSDARG00000024204)<br>mcm2 (ENSDARG00000102798)<br>mcm5 (ENSDARG00000019507)<br>mcm4 (ENSDARG00000040041)<br>hells (ENSDARG00000057738)<br>mcm6 (ENSDARG00000057683)
	Single-stranded DNA binding (GO:0003697)	5	0.081967	5.34×10^{-5}	mcm3 (ENSDARG00000024204)<br>mcm2 (ENSDARG00000102798)<br>mcm5 (ENSDARG00000019507)<br>mcm4 (ENSDARG00000040041)<br>mcm6 (ENSDARG00000057683)
	Nucleotide binding (GO:0000166)	15	0.009536	0.003745	vars1 (ENSDARG00000044575)<br>mcm3 (ENSDARG00000024204)<br>mcm2 (ENSDARG00000102798)<br>msh6 (ENSDARG00000104541)<br>mcm5 (ENSDARG00000019507)<br>sephs1 (ENSDARG00000058292)<br>mcm4 (ENSDARG00000040041)<br>tuba1b (ENSDARG00000045367)<br>hells (ENSDARG00000057738)<br>pold1 (ENSDARG00000027689)<br>aldh18a1 (ENSDARG00000099579)<br>myo1d (ENSDARG00000036863)<br>mcm6 (ENSDARG00000057683)<br>tuba1a (ENSDARG00000001889)<br>si:ch211-114n24.6 (ENSDARG00000036700)
	Structural constituent of cytoskeleton (GO:0005200)	3	0.085714	0.004203	tuba1b (ENSDARG00000045367)<br>tuba1a (ENSDARG00000001889)<br>si:ch211-114n24.6 (ENSDARG00000036700)
	Chromatin binding (GO:0003682)	5	0.025381	0.008519	phf13 (ENSDARG00000102023)<br>mcm3 (ENSDARG00000024204)<br>mcm2 (ENSDARG00000102798)<br>dnmt1 (ENSDARG00000030756)<br>mcm4 (ENSDARG00000040041)
	**Cellular component**				
	MCM complex (GO:0042555)	5	0.384615	1.98×10^{-8}	mcm3 (ENSDARG00000024204)<br>mcm2 (ENSDARG00000102798)<br>mcm5 (ENSDARG00000019507)<br>mcm4 (ENSDARG00000040041)<br>mcm6 (ENSDARG00000057683)
	Nucleus (GO:0005634)	23	0.00552	0.072182	phf13 (ENSDARG00000102023)

				mcm3 (ENSDARG00000024204)<br>mcm2 (ENSDARG00000102798)<br>mcm5 (ENSDARG00000019507)<br>dnmt1 (ENSDARG00000030756)<br>sephs1 (ENSDARG00000058292)<br>mcm4 (ENSDARG00000040041)<br>zgc:77262 (ENSDARG00000044545)<br>gadd45ga (ENSDARG00000019417)<br>chaf1b (ENSDARG00000056473)<br>nfil3-6 (ENSDARG00000087188)<br>mybl2b (ENSDARG00000032264)<br>cdca7a (ENSDARG00000077620)<br>znf367 (ENSDARG00000021086)<br>hells (ENSDARG00000057738)<br>crema (ENSDARG00000023217)<br>ipo8 (ENSDARG00000058159)<br>pold1 (ENSDARG00000027689)<br>tcf19l (ENSDARG00000040036)<br>ankrd1b (ENSDARG00000076192)<br>si:ch211-89o9.4 (ENSDARG00000093237)<br>mcm6 (ENSDARG00000057683)<br>ccnb1 (ENSDARG00000051923)
Cyclin B1-CDK1 complex (GO:0097125)	1	1	0.072182	ccnb1 (ENSDARG00000051923)
Mutsalpha complex (GO:0032301)	1	0.5	0.082892	msh6 (ENSDARG00000104541)
CAF-1 complex (GO:0033186)	1	0.333333	0.099312	chaf1b (ENSDARG00000056473)
Delta DNA polymerase complex (GO:0043625)	1	0.25	0.11017	pold1 (ENSDARG00000027689)
Mitochondrial matrix (GO:0005759)	2	0.030303	0.120492	ccnb1 (ENSDARG00000051923)<br>alas1 (ENSDARG00000021059)
**Biological process**				
Pre-replicative complex assembly involved in nuclear cell cycle DNA replication (GO:0006267)	5	0.555555556	4.38×10^{-9}	mcm3 (ENSDARG00000024204)<br>mcm2 (ENSDARG00000102798)<br>mcm5 (ENSDARG00000019507)<br>mcm4 (ENSDARG00000040041)<br>mcm6 (ENSDARG00000057683)
Double-strand break repair via break-induced replication (GO:0000727)	5	0.384615385	2.22×10^{-8}	mcm3 (ENSDARG00000024204)<br>mcm2 (ENSDARG00000102798)<br>mcm5 (ENSDARG00000019507)<br>mcm4 (ENSDARG00000040041)<br>mcm6 (ENSDARG00000057683)
DNA replication initiation (GO:0006270)	5	0.238095238	2.29×10^{-7}	mcm3 (ENSDARG00000024204)<br>mcm2 (ENSDARG00000102798)<br>mcm5 (ENSDARG00000019507)<br>mcm4 (ENSDARG00000040041)<br>mcm6 (ENSDARG00000057683)
DNA replication (GO:0006260)	6	0.068965517	8.38×10^{-6}	mcm3 (ENSDARG00000024204)<br>mcm2 (ENSDARG00000102798)<br>mcm5 (ENSDARG00000019507)<br>mcm4 (ENSDARG00000040041)<br>pold1 (ENSDARG00000027689)<br>mcm6 (ENSDARG00000057683)
Mitotic DNA replication initiation (GO:1902975)	3	0.6	8.38×10^{-6}	mcm3 (ENSDARG00000024204)<br>mcm2 (ENSDARG00000102798)<br>mcm4 (ENSDARG00000040041)

Mitotic cell cycle (GO:0000278)	6	0.06521739 1	9.56×10^{-6}	mcm5 (ENSDARG00000019507) tuba1b (ENSDARG00000045367) chaf1b (ENSDARG00000056473) mybl2b (ENSDARG00000032264) tuba1a (ENSDARG00000001889) si:ch211-114n24.6 (ENSDARG00000036700)
DNA unwinding involved in DNA replication (GO:0006268)	3	0.27272727 3	9.75×10^{-5}	mcm2 (ENSDARG00000102798) mcm4 (ENSDARG00000040041) mcm6 (ENSDARG00000057683)

References

1. Cock, P.J.A.; Fields, C.J.; Goto, N.; Heuer, M.L.; Rice, P.M. The Sanger FASTQ file format for sequences with quality scores, and the Solexa/Illumina FASTQ variants. *Nucleic Acids Research* **2010**, *38*, 1767-1771, doi:10.1093/nar/gkp1137.
2. Kim, D.; Landmead, B.; Salzberg, S.L. HISAT: a fast spliced aligner with low memory requirements. *Nature Methods* **2015**, *12*, 357-U121, doi:10.1038/nmeth.3317.
3. Langmead, B.; Salzberg, S.L. Fast gapped-read alignment with Bowtie 2. *Nature Methods* **2012**, *9*, 357-359, doi:10.1038/nmeth.1923.
4. Li, B.; Dewey, C.N. RSEM: accurate transcript quantification from RNA-Seq data with or without a reference genome. *BMC Bioinformatics* **2011**, *12*, 323, doi:10.1186/1471-2105-12-323.
5. Love, M.I.; Huber, W.; Anders, S. Moderated estimation of fold change and dispersion for RNA-seq data with DESeq2. *Genome Biology* **2014**, *15*, 550, doi:10.1186/s13059-014-0550-8.
6. Abdi, H. The Bonferonni and Šidák Corrections for Multiple Comparisons. *Encyclopedia of measurement and statistics* **2007**, *1*, 1-9.
